# MLPA as a genetic assay for the prenatal diagnosis of common aneuploidy: the first Egyptian experience

**DOI:** 10.1186/s43141-022-00402-8

**Published:** 2022-07-28

**Authors:** Ola M. Eid, Maha M. Eid, Marwa Farid, Rania M. A. Abdel Kader, Rana Mahrous, Sara H. El-Dessouky

**Affiliations:** 1 Departments of Human Cytogenetics, National Research Centre, El Bohouth Street, Dokki, Cairo 12311 Egypt; 2 Prenatal Diagnosis & Fetal Medicine, National Research Centre, El Bohouth Street, Dokki, Cairo 12311 Egypt

**Keywords:** MLPA, FISH, Prenatal diagnosis, Aneuploidy

## Abstract

**Background:**

The prenatal diagnosis of syndromes caused by chromosomal abnormality is a long-established part of obstetric care. Several DNA-based molecular approaches have provided rapid prenatal diagnosis of of cytogenomic abnormalities. MLPA has become available for rapid aneuploidy detection of the most common chromosome abnormalities.

**Objectives:**

The aim of this study is to introduce the MLPA technique as a method for the prenatal detection of aneuploidy in Egypt by its validation compared to the FISH technique.

**Methods:**

Fifty AF samples were collected for this study and were subjected to MLPA and FISH assays to detect the most common prenatal chromosomal abnormality.

**Results and conclusions:**

Our study confirmed previous reports that MLPA is analogous to FISH for detecting common aneuploidies and could be a quick and dependable tool for prenatal diagnosis. Therefore, initial prompt testing of AF samples for the copy number of the most common occurring aneuploidies is recommended.

## Background

The prenatal diagnosis of syndromes caused by chromosomal abnormality is a long-established part of obstetric care [1]. The conventional methods in prenatal diagnosis are invasive amniocentesis of amniotic fluid and chorionic villus sampling procedures, noninvasive maternal serum screening, and high-resolution ultrasound examination. Advanced maternal age for increased risk of Down syndrome, abnormal maternal serum screening, abnormal ultrasound findings, family history of chromosomal or genetic disorders, history of spontaneous abortion, and integrated maternal serum fetal DNA sequencing for aneuploidy detection are the major clinical indications for prenatal diagnosis [2].

Low analytical resolution and long turnaround time are the limitations of routine prenatal conventional cytogenetic analysis. Several DNA-based molecular approaches have provided rapid prenatal diagnosis of cytogenomic abnormalities [2]. These approaches have dramatically minimized turnaround times from 1 to 2 weeks to 1 to 2 days [1]. For example, fluorescence in situ hybridization (FISH) allows the rapid detection of locus-specific numerical aberrations [3]. Another example is quantitative fluorescence-polymerase chain reaction (QF-PCR) used for the prenatal diagnosis of fetal aneuploidies [4].

In developed countries, multiplex ligation-dependent probe amplification (MLPA) has become available for rapid aneuploidy detection of the most common chromosome abnormalities (aneuploidies of chromosomes X, Y, 13, 18, and 21). MLPA was first described in 2002 by Shouten et al. MLPA is a multiplex PCR method detecting abnormal copy numbers of up to 50 different genomic DNA or RNA sequences, which can distinguish sequences differing in only one nucleotide [5]. It only requires a thermocycler and capillary electrophoresis equipment. Up to 96 samples can be handled simultaneously, with results available within 24 h. The inclusion of MLPA in clinical settings significantly increases the detection rate of many genetic disorders [6]. This technology has considerable advantages in that it is highly versatile in its applications, malleable in its target loci, highly automated, appropriate for high-throughput testing, competent, and cost effective.

This study aimed to introduce the MLPA technique as a method for the prenatal detection of aneuploidy, the most common prenatal chromosomal abnormality, in Egypt by its validation compared to the FISH technique.

## Methods

This study was conducted at our institute and approved by its Medical Ethical Committee. Informed written consent was obtained from the study participants. Fifty pregnant women were included in this study. Their age at the time of sampling ranged from 30 to 40 years. They were suspected of having fetal aneuploidies from detailed ultrasound scanning. Amniocentesis was carried on an outpatient basis ~16 weeks of gestation (second trimester) after the parents’ acceptance. Clear AF (15–20 cc) was collected and sent immediately for laboratory studies.

### FISH analysis

Half of the AF sample was prepared for the FISH examination. The sample was centrifuged and subjected to hypotonic solution for 2 to 3 h. The sample was processed by fixation and slide preparation. FISH was done according to the Pinkel et al. [7] and manufacturer’s instructions of the probe. FISH probes for detecting common aneuploidies (13, 18, 21, and XY) were used. All FISH probes were commercially available (Vysis FISH probes; Abbott Molecular, Inc., USA). The hybridized probe fluoresces with moderate to bright intensity in interphase nuclei that appear as distinct signals. A total of 200 interphase cells were examined per sample. One sample was subjected to examination by the Metasystem CEP 2 FISH probe.

### MLPA assay

The other half of the AF sample was subjected to DNA extraction using either the QIAamp DNA Mini Kit or the PAXgene Blood DNA Kit (Germany) according to the manufacturer’s instructions. The quality and quantity of the DNA samples were determined using a NanoDrop spectrophotometer.

MLPA assay was performed using SALSA MLPA Probemix P095 aneuploidy according to the manufacturer’s instructions (MRC-Holland, The Netherlands). This probemix contains 36 MLPA probes: 8 probes for each chromosome 13, 18, 21, and X and 4 probes for the Y chromosome. DNA denaturation and overnight MLPA probemix hybridization steps were followed by probe ligation and amplification on the following day. The amplified products were separated using an ABI 3500 Genetic Analyzer (Applied Biosystems, USA). The results were interpreted using Coffalyser.Net software (MRC-Holland). Ratios of < 0.75, 0.75 to 1.30, and > 1.3 were considered to indicate deletion, normal, and duplication, respectively.

## Results

**Fig. 1:**
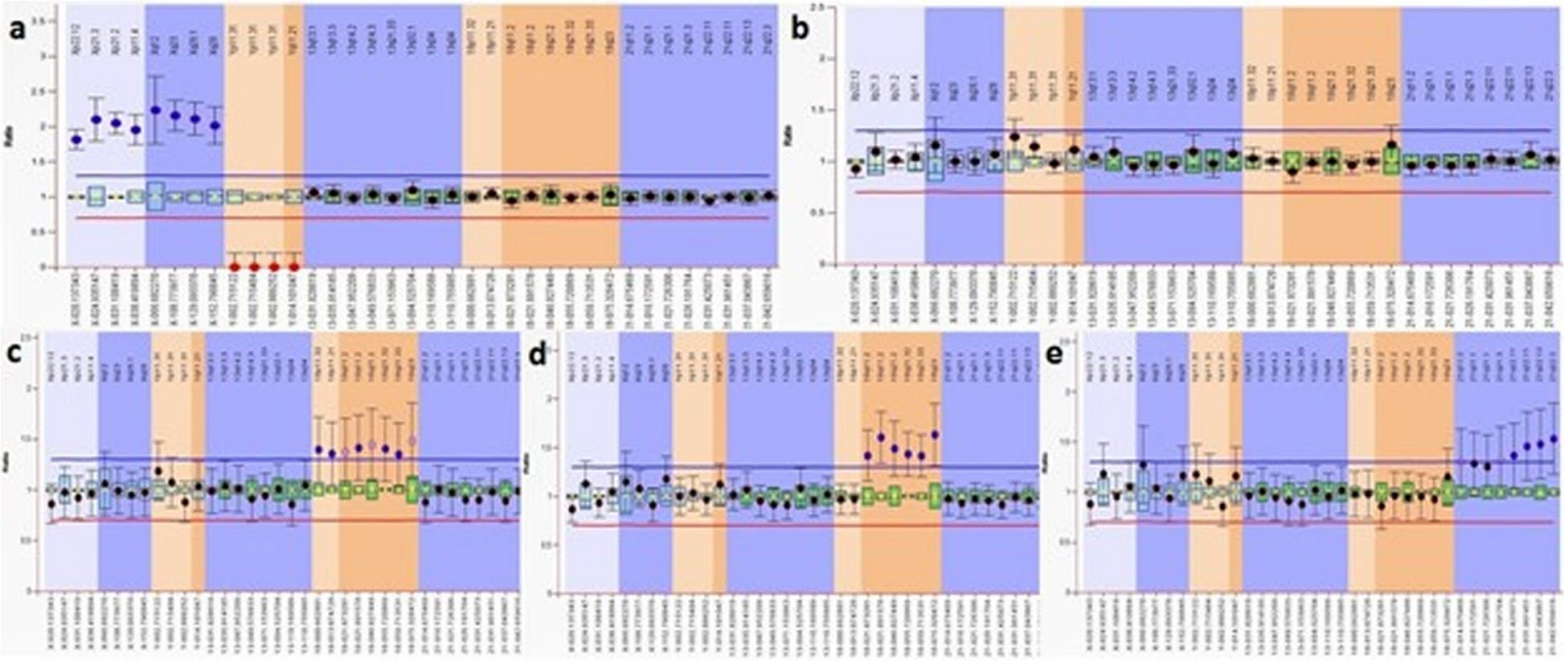
Ratio charts of the MLPA results for some AF samples using SALSA MLPA Probemix P095 aneuploidy. Probe ratios of < 0.7 (red line) or > 1.3 (blue line) are usually regarded as indicative of a deletion or duplication, respectively. **a** XX sample with no trisomy for 13, 18, and 21; **b** XY sample with no trisomy for 13, 18, and 21; **c** XY sample having trisomy 18; **d** XY sample having trisomy 18q; **e** XY sample having trisomy 21

In this study, 50 AF samples were included. The MLPA and FISH results are summarized in Table 1. Two samples were not subjected to either MLPA or FISH assays because their DNA concentration was too low to be examined by MLPA (<10 ng/μl). Figure 1 shows the ratio charts of the MLPA results for some AF samples using SALSA MLPA Probemix P095 aneuploidy.

**Table 1:** Summary of the MLPA and FISH results

	MLPA result	MLPA gender	FISH result	FISH gender
1	NTD	XX	NTD	XX
2	T18	XY	T18	XY
3	NTD	XX	NTD	XX
4	NTD	XY	NTD	XY
5	NTD	XY	NTD	XY
6	NTD	XY	NTD	XY
7	NTD	XX	NTD	XX
8	NTD	XX	NTD	XX
9	T18	XY	T18	XY
10	T18q	XY	T18	XY
11	T18	XY	T18	XY
12	NTD	XX	NTD	XX
13	NTD	XY	NTD	XY
14	NTD	XY	NTD	XY
15	NTD	XY	NTD	XY
16	NTD	XX	NTD	XX
17	NTD	XX	NTD	XX
18	NTD	XX	NTD	XX
19	T21	XY	mos T21 70%	XY
20	T21	XY	mos T21 70%	XY
21	T21	XX	mos T21 60%	XX
22	NTD	XX	NTD	XX
23	NTD	XY	NTD	XX
24*	NTD	XY	T13,T18,T21	XXY
25	T21	XY	mos T21 50%	XY
26	T21	XX	mos T21	XX
27	NTD	XY	NTD	XY
28	NTD	XY	NTD	XY
29	NTD	XY	NTD	XY
30	NTD	XY	NTD	XY
31	NTD	XY	NTD	XY
32	NTD	XY	NTD	XY
33	NTD	XX	NTD	XX
34	T18	XY	T18	XY
35	T18	XY	T18	XX
36	NTD	XY	NTD	XY
37	NTD	XY	NTD	XY
38	Not Done	Not Done	Not Done	Not Done
39	NTD	XY	NTD	XY
40	NTD	XY	NTD	XY
41	NTD	XX	NTD	XX
42	T21	XX	mos T21	XX
43	T21	XX	mos T21	XY
44	NTD	XY	NTD	XY
45	Not Done	Not Done	Not Done	Not Done
46	NTD	XY	NTD	XY
47	NTD	XX	NTD	XX
48	NTD	XX	NTD	XX
49	NTD	XY	NTD	XY
50	NTD	XX	NTD	XX

*NTD* no trisomy detected. *T2 was detected in this sample and with the other trisomies, indicating triploidy

**Fig. 2:**
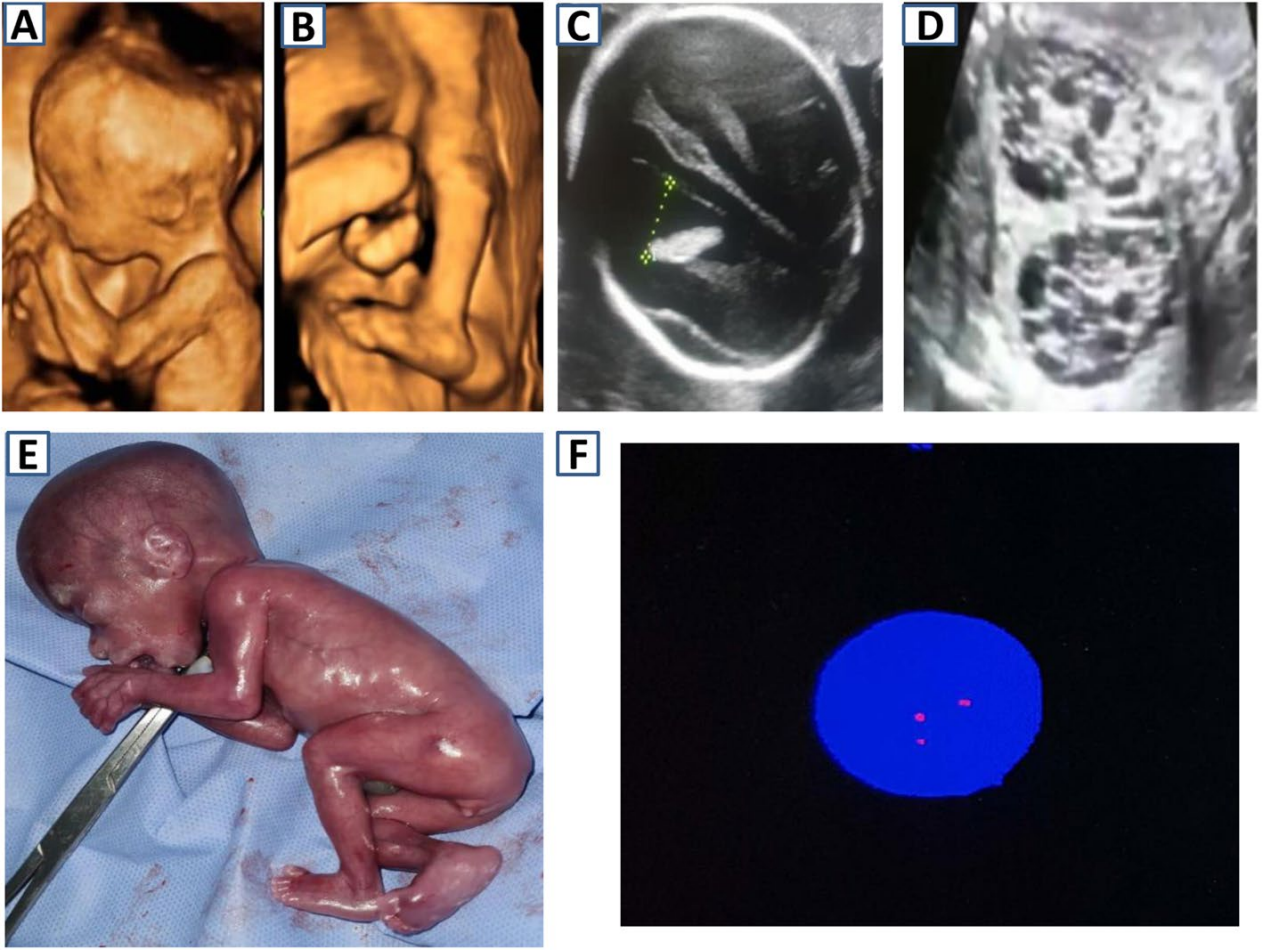
A case of trisomy 18 detected in the second trimester. **A** Three-dimensional surface rendering of the fetal face showing low set ears and clenched hands. **B** Three-dimensional surface rendering mode of the lower extremities showing bilateral rocker-bottom feet. **C** Axial transventricular plane of the fetal head showing ventriculomegaly. **D** Coronal ultrasound image of a multicystic dysplastic kidney (MCDK) showing several subcortical small cysts. **E** Postmortem image confirms the presence of the above findings. **F** FISH technique showing trisomy 18

**Fig. 3:**
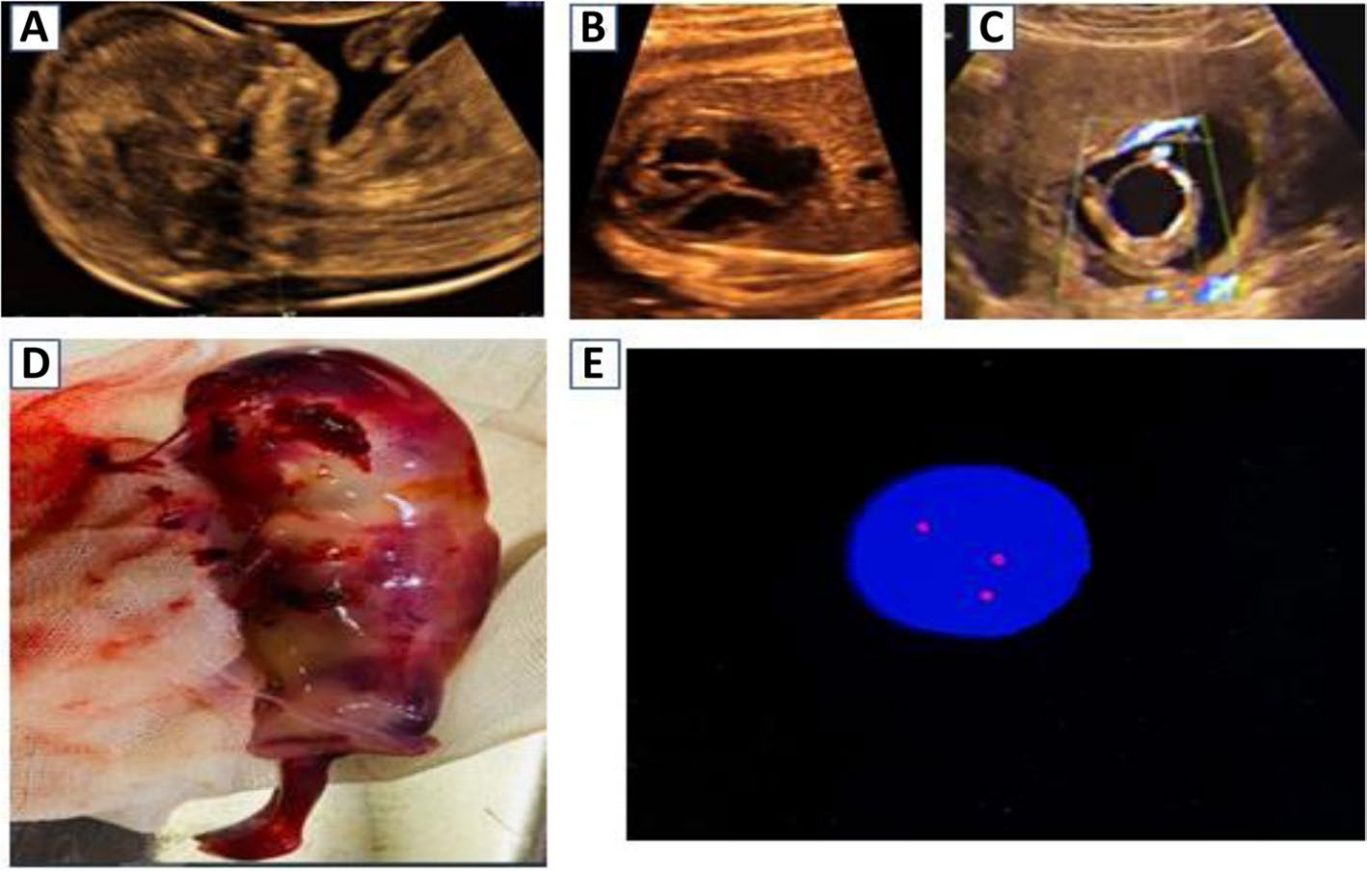
A case of trisomy 21 detected in the first trimester. **A** Midsagittal view of the fetal face showing hypoplastic nasal bone and increased nuchal translucency. **B** Transverse views of the fetal chest at the level of the four chambers showing an atrioventricular septal defect. **C** Axial view of the fetal pelvis showing megacystis and single umbilical artery. **D** A postmortem image confirms the presence of the above findings. **E** FISH technique showing trisomy 21

Of the 48 studied samples, 6 samples (12.5%) showed trisomy 18 (Fig. 2), and 7 samples (~14.6%) showed trisomy 21 (Fig. 3) by both MLPA and FISH. One sample of trisomy 18 showed T18q only by MLPA. No sample showed sole trisomy 13 by either technique. The MLPA probe ratios of the trisomies ranged from 1.35 to 1.75.

**Fig. 4:**
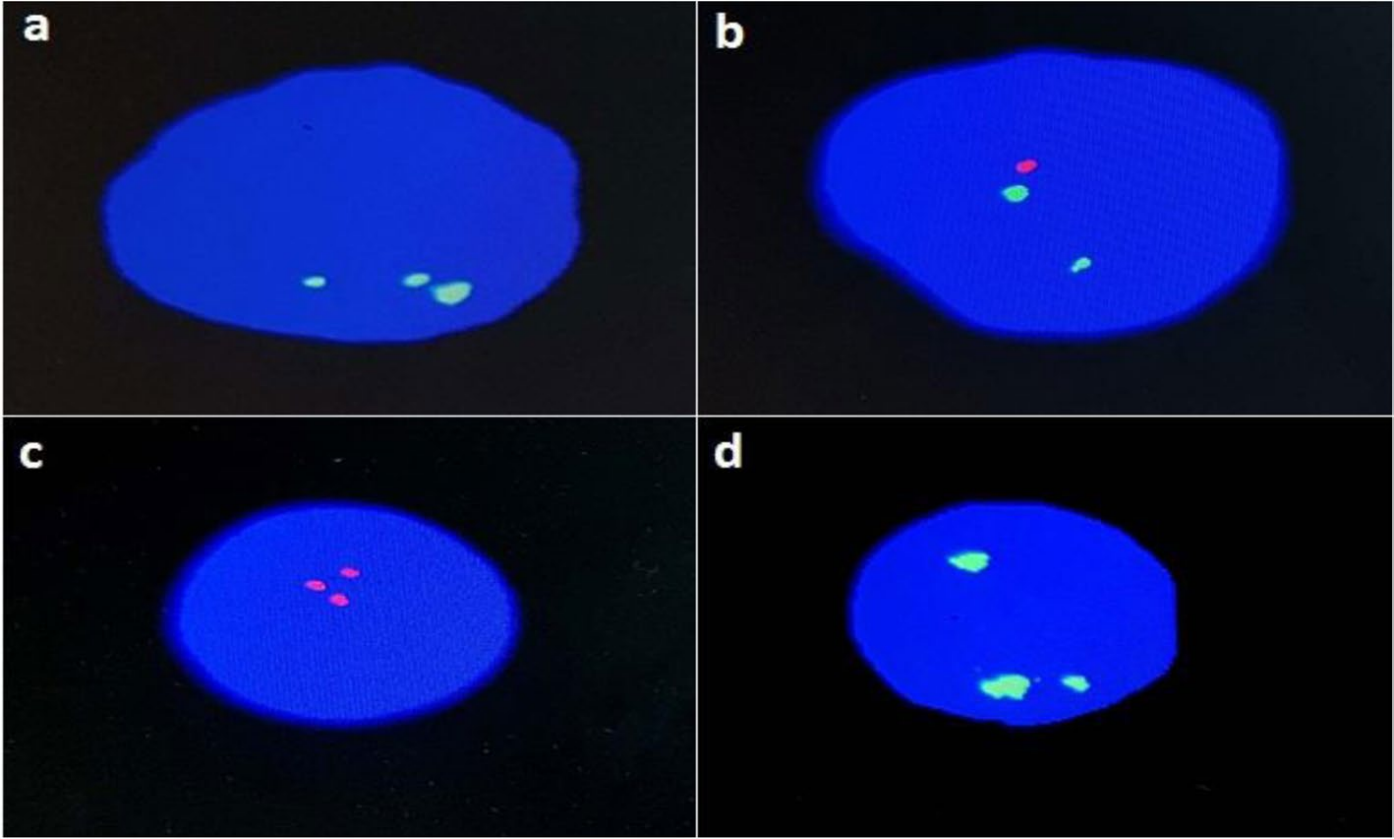
FISH for the triploidy sample: **a** using CEP 2 (Metasystem) spectrum green showing three signals indicating the triploidy; **b** using CEP X,Y probe showing two green signals for the X chromosome and one red signal for the Y chromosome; **c** using LSI 21 showing three red signals; **d** using LSI 13 showing three green signals

**Fig. 5:**
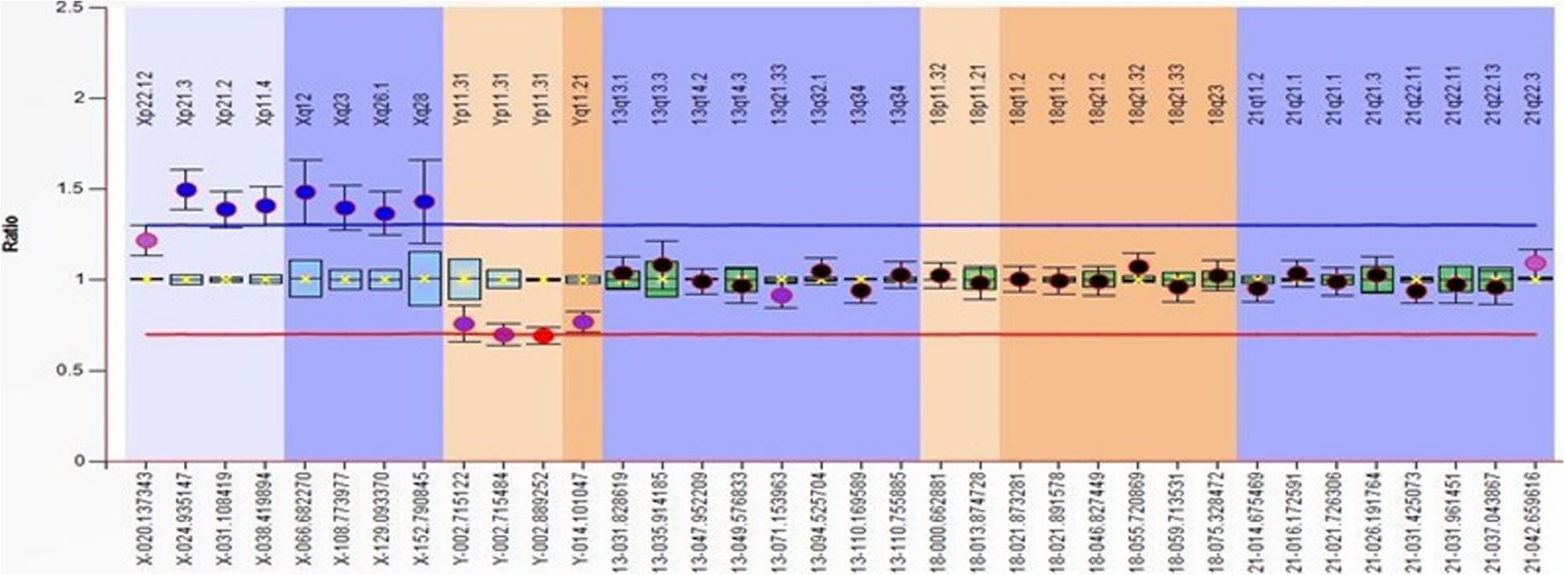
Ratio charts of the MLPA results for the triploidy sample. The MLPA probe ratio showed duplication of ~1.36 for X probes and ~0.73 for Y probes, detecting signals for the Y chromosome and indicating the XXY sample

One sample was suspected of having triploidy depending on ultrasonography findings. A FISH study using centromere 2 spectrum green was done and showed three signals denoting the triploidy. Also, CEP X, Y probe and LSI 21 and 13 were used and showed XXY and trisomies 21 and 13, respectively (Fig. 4). In contrast, the MLPA study could not detect any trisomies in this case. However, the MLPA probe ratio showed duplication of ~1.36 for X probes and ~0.73 for Y probes, detecting signals for the Y chromosome and indicating the XXY sample (Fig 5).

Nineteen of the 48 studied samples (~39.5%) were for XX female fetuses, and 28 samples (~60.4%) were for XY male fetuses by both MLPA and FISH. The MLPA probe ratios of the X probes ranged from 1.67 to 2.56, whereas the Y probe ratios were between 0.75 and 1.30 for the males and were 0 for females.

## Discussion

Fifty AF samples were collected for this study. However, only 48 AF samples were studied. Two samples were not subjected to either MLPA or FISH assays, because their DNA concentration was too low to be examined by MLPA (<10 ng/μl). This could be explained by either the sample was withdrawn earlier than 16 weeks of gestation or because it was extracted using the QIAamp DNA Mini Kit (which yields lesser DNA concentrations) than the PAXgene Blood DNA Kit (which generally yields higher DNA concentrations), suggested to be more suitable for AF samples.

The availability of a rapid and economical assay to detect aneuploidy, the most common prenatal chromosomal abnormality for high-risk pregnancies, is the reason for the employment of MLPA. Slater et al. conducted a blind prospective trial using MLPA prenatal detection of common aneuploidies (13, 18, 21, X, and Y) on 492 amniotic samples referred for routine testing [1]. There were no failed tests. The difference in distributions of normal and aneuploid samples clearly identified all 17 autosomal aneuploid patients. Sex determination was also 100% accurate and included a single case of monosomy X. In 2008 and 2009, Gerdes et al. [8] and Van Opstal et al. [9] evaluated MLPA performance as a method for the rapid prenatal diagnosis of common aneuploidies on a total of 3925 and 4000 samples, respectively. They concluded that MLPA is a reliable method that can replace FISH and karyotyping as large-scale testing for rapid aneuploidy diagnosis. Hamidah et al. applied the MLPA technique to detect aneuploidies in AF samples from 25 pregnant women versus the QF-PCR method [10]. Conclusive results were obtained, including one case with maternal cell contamination. All results agreed with that of the QF-PCR.

In this study, 48 AF samples were screened for 13, 18, and 21 aneuploidy using MLPA and FISH techniques. Six samples (12.5%) showed trisomy 18 by both MLPA and FISH; one sample of the trisomy 18 showed T18q only by MLPA and was detected as full trisomy by FISH. Seven samples (~14.6%) showed trisomy 21 by both MLPA and FISH. The FISH study detected mosaicism of ~60%. Mosaicism indicated the presence of two different cell lines in one individual. MLPA analysis is expected to detect a high level of chromosomal mosaicism, giving the average copy number per cell. Detection of no abnormality by the MLPA assay cannot eliminate the possibility of low-level mosaicism. However, the definition of low mosaicism, or the level at which MLPA could not detect abnormalities, has differed between studies and remains controversial. Nevertheless, the reported mosaicism levels detected by MLPA ranged between 20 and 30%. Moreover, true mosaicism that is clinically relevant is associated with high levels and is more likely to be identified by MLPA [11–13]. Despite the mosaicism detected by FISH in this study, MLPA could perfectly detect the aneuploidy in the samples.

In this study, one sample showed triploidy detected by FISH only. Generally, the MLPA assay cannot detect female triploidy but has the variable capability to detect male triploidy. However, triploidies could be suspected by the presence of fetal ultrasound abnormalities, and the assay of choice is then selected, such as QF-PCR [11, 13, 14]. Nonetheless, sex determination for this case was accurately detected by MLPA as XXY and was confirmed by FISH.

Finally, all MLPA results were conclusive and in concordance with FISH results, with 100% sensitivity and 100% specificity, except for the one case of triploidy. There were no false-negative or false-positive results. Sex determination was also 100% accurate.

## Conclusion

This study confirmed that MLPA is analogous to FISH for detecting common aneuploidies. So, MLPA could be a quick and dependable tool for prenatal diagnosis in Egypt with its significant advantages as it is highly versatile in its applications, malleable in its target loci, highly automated, short turnaround time, appropriate for high-throughput testing, competent, and cost effective. Therefore, initial prompt testing of AF samples for the copy number of the most common occurring aneuploidies is recommended. Moreover, because of high effectiveness of MLPA assays in postnatal diagnosis of single gene-disorders, the normal samples could be further tested based on the clinical preselection by MLPA using probemixes for the most common microdeletion syndromes together with all subtelomeric regions

## Data Availability

The authors confirm that the data supporting the findings of this study are available within the article.
